# The Book World of Medicine and Science

**Published:** 1895-05-04

**Authors:** 


### THE BOOK WORLD OF MEDICINE AND SCIENCE.

ENLARGEMENT OF THE PROSTATE. By C. W. MANSELL MOULLIN, M.A., M.D., F.R.C.S. (London: H. K. Lewis, 1894. Price 6s.)

This is a careful and thorough book, and one which is sure to be of great use to those who have to treat and to advise patients suffering from this trying disorder. The author points out that during recent years a large amount of new knowledge has been added to our store in regard to this disease, and that new methods have been introduced which make such cases more amenable to treatment than they ever were before, and, although no such excuse was needed for writing so sound a book, he puts that forward as a reason for its production. After an account of the normal and the diseased prostate and of the causes of its enlargement, the effects of this malady upon the bladder and the kidneys are considered. It is stated that the injurious effect of prostatic enlargement upon the kidneys is not due entirely to the back pressure of the retained urine, but that by reflex irritation a considerable congestion of the kidneys is kept up which gradually spoils their efficiency. The importance of arriving at a true conclusion regarding the cause of renal degeneration is insisted on. The signs of this degeneration in its early stages are vague and ill-defined. The evils attending the habitual use of catheters are well and forcibly pointed out, and the picture of the "catheter life" entered into by many at a comparatively early age is not a pleasant one. It must be continued for the rest of life; sooner or later, the constant passage of the instrument leads to congestion and irritability of the neck of the bladder; micturition becomes more frequent, so that there may be no rest night or day, and, as the gland continues to enlarge, the introduction of the catheter becomes more difficult, and when congestion supervenes may be almost impossible. In the ordinary course the catheter causes cystitis. "The comparative comfort so often (but not always) seen at the beginning of catheter life is very apt to throw into the background the much darker picture of what the future will be." After this we are quite prepared to find that Dr. Mansell Moullin advocates operative treatment for the removal of a disease which has such a sad future in store for those who suffer from it. The choice lies, he says, between the habitual employment of a catheter; removal of the obstruction by operation; and, when nothing else is available, the formation of another route of exit or castration. In all ordinary cases, unless the disease is already advanced or some special feature is present, catheterism is to be preferred. On the other hand, when certain anatomical conditions which interfere with the function of the bladder are present, or when, though the catheter may have succeeded at first, the patient is beginning to fail, the bladder is losing its power, the amount of residual urine is increasing, or inflammation is setting in, prostatectomy should be performed before it is too late, or as a last resource castration.

ARTIFICIAL FEEDING AND FOOD DISORDERS OF INFANTS. By W. B. CHEADLE, M.D., F.R.C.P. Third Edition. 1894. (London: Smith, Elder, and Co. Pp. 248. Price 5s.)

This well-known book is an interesting and important work, in view of the growing prevalence and even necessity of the artificial rearing of infants. We are of those who doubt the possibility of imposing entirely new conditions on the method of development of any class of animals without producing injury in some direction or another. Yet the circumstances amid which a yearly increasing number of children are introduced into this world are such that artificial feeding is the only form of nourishment open to them. Herein then is the importance of this work which puts into acceptable and usable form a sort of knowledge which all men of observation and experience do in time work out for themselves in a more or less rough and ready manner. A careful account is given of the scientific principles upon which artificial feeding should be based, and naturally milk stands out pre-eminent as the proper basis of infants' foods, and descriptions are given of the various devices for producing from cows' milk a milk acceptable to babes, and for tiding over times when, may be as a temporary difficulty, children fail to digest milk. Other forms of food are also considered, but in all cases caution is given to beware of the deficiency which is so apt to exist in them of anti-scorbutic elements. The diseases which result from errors of diet are given, divided into two classes. The book is full of useful hints, and it is to be praised not only for its accuracy but for its attention to detail.

